# Association between DNA Methylation in the miR-328 5’-Flanking Region and Inter-individual Differences in miR-328 and BCRP Expression in Human Placenta

**DOI:** 10.1371/journal.pone.0072906

**Published:** 2013-08-21

**Authors:** Jumpei Saito, Takeshi Hirota, Shinji Furuta, Daisuke Kobayashi, Hiroshi Takane, Ichiro Ieiri

**Affiliations:** 1 Department of Clinical Pharmacokinetics, Graduate School of Pharmaceutical Sciences, Kyushu University, Fukuoka, Japan; 2 Department of Pharmacy, Tottori University Hospital, Yonago, Japan; University of Barcelona, Spain

## Abstract

MicroRNA (miRNA) are non-coding small RNA that regulate gene expression. MiR-328 is reported to influence breast cancer resistance protein (BCRP) expression in cancer cells. As a large inter-individual difference in BCRP levels is observed in various human tissues, the contribution of miR-328 to these differences is of interest. We hypothesized that DNA methylation in the miR-328 promoter region is responsible for the difference in miR-328 levels, leading to inter-individual variability in BCRP levels in human placenta. The association between placental miR-328 and BCRP levels was analyzed, and then DNA methylation in the miR-328 5'-flanking region and regulatory mechanisms causing inter-individual differences in miR-328 and BCRP levels were examined. MiR-328 expression was significantly correlated with BCRP mRNA (*Rs* = -0.560, *P* < 0.01) and protein (*Rs* = -0.730, *P* < 0.01) levels. It was also up-regulated by the demethylating agent 5-aza-2’-deoxycytidine in BCRP-expressing cells. Luciferase assays with differentially methylated reporter constructs indicated that methylation in the miR-328 5’-flanking region including a predicted CpG island remarkably decreased transcriptional activity compared to that in unmethylated constructs. We selected CCAAT/enhancer binding protein α (C/EBPα), located within the predicted CpG island, by *in silico* analysis. To elucidate the role of C/EBPα in miR-328 expression, a chromatin immunoprecipitation assay, promoter deletion analysis, and electrophoretic mobility shift assay (EMSA) were performed. C/EBPα-binding site-truncated constructs showed significantly decreased promoter activity, and EMSA indicated that the C/EBPα-binding sites were located in the CpG island. Finally, the methylation patterns of several CpG dinucleotides proximal to two C/EBPα-binding sites in the miR-328 5’-flanking region were correlated negatively with miR-328 levels, and positively with BCRP levels in human placental samples. These results suggest that methylation patterns in the miR-328 5’-flanking region are involved in the inter-individual difference in BCRP levels in human placenta.

## Introduction

Breast cancer resistance protein (BCRP) is constitutively expressed in normal human tissues including the intestine, liver, blood–brain barrier, breast, and placenta, as well as tumor tissues, and is involved in multidrug resistance because BCRP acts as an efflux transporter of anti-cancer drugs [1]. Previous studies have shown that expression levels of BCRP in human placentas were profoundly affected by genetic polymorphisms. Several non-synonymous single nucleotide polymorphisms (SNPs) in the *BCRP* gene (*ABCG2*) have been associated with reduced BCRP transport activity [2–7]. Based on these observations, the genetic variation in *BCRP* is considered to contribute to the inter-individual variability in the pharmacokinetics of BCRP substrate drugs. However, the mechanisms causing inter-individual differences in BCRP levels are not fully understood. Besides SNPs, transcription factors [8–14], epigenetic factors [15–19] and microRNAs (miRNAs) [20] play important roles in the regulation of BCRP expression.

MiRNA are small noncoding RNAs, approximately 20-25 nucleotides in length, which target the 3’-untranslated region (3’-UTR) of mRNA specifically to prevent translation of mRNA or to degrade mRNA [20]. MiRNA expression levels in normal tissues show large inter-individual variability [21,22]. MiRNA also influence the expression of drug-metabolizing enzymes [23–27] and drug transporters [20,28–34], suggesting that the variations in miRNA expression contribute to the inter-individual differences in drug response. It was reported that miR-328 influenced drug efflux by repressing BCRP mRNA expression in breast cancer cells [20]. Other studies demonstrated that miR-328 plays an inhibitory role in the proliferation of cancer cell lines and indicated that the suppression of miR-328 expression provides an advantage for tumor growth [35–39]. Studies have also showed that miR-328 is important in regulating BCRP expression, but little is known about its contribution to the variability in BCRP levels among individuals.

One-third of all human miRNA may be regulated by DNA methylation in CpG islands upstream [40]. In fact, previous reports showed that miR-127, miR-124a, and miR-34 were down-regulated by epigenetic events such as DNA methylation [41,42]. It was also reported that hypermethylation in the miR-328 upstream region in urine specimens from bladder cancer patients was associated with tumor grade, stage and prognosis, and the reduced expression of miR-328 itself [43].

In this study, we focused on the inter-individual variability in BCRP and miR-328 levels in the human placenta. We analyzed the relationship between miR-328 and BCRP mRNA and protein levels. Furthermore, we evaluated the importance of methylation patterns to individual miR-328 levels.

## Results

### The association between miR-328 and BCRP levels in the human placenta

**Figure 1:**
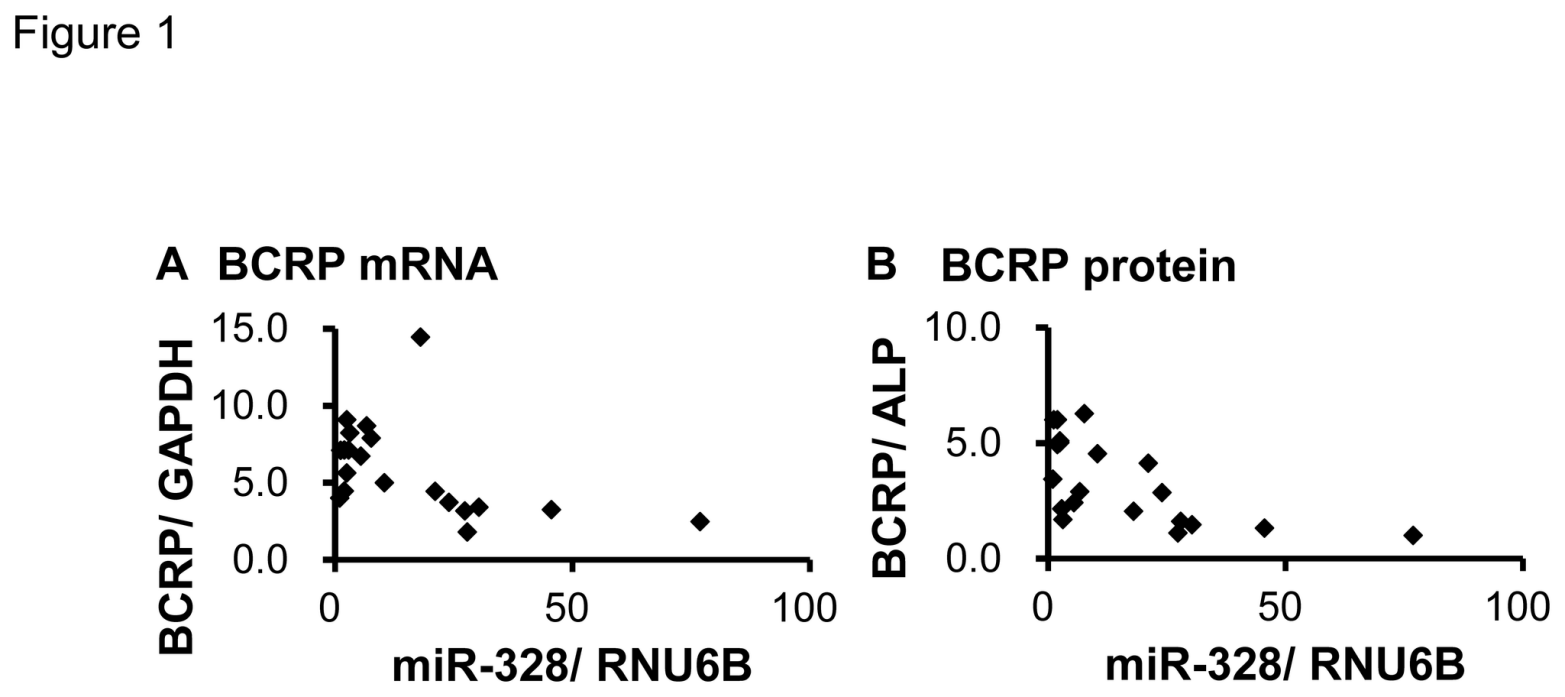
Correlation between miR-328 and BCRP mRNA (A) or protein (B) levels in human placentas (n = 20). Each BCRP mRNA, BCRP protein and miR-328 level was normalized by the minimum value.

The relationship between miR-328 and BCRP levels (mRNA and protein) was analyzed using 20 human placental samples. It was reported that some SNPs (i.e., C421A, G34A and C376T genotypes) significantly impacted on BCRP function [2–4]. To eliminate the influences of *BCRP* genetic variants, we selected samples with wild-type homozygotes for C421A, G34A and C376T. BCRP mRNA and protein levels both showed a significantly negative correlation with miR-328 levels (mRNA, *Rs* = -0.560, *P* = 0.00526; protein, *Rs* = -0.730, *P* = 0.000132) (**Figure 1**). Furthermore, an over 80-fold inter-individual difference was observed in miR-328 levels in the human placentas.

### A single CpG island in the miR-328 5’-flanking region

**Figure 2:**
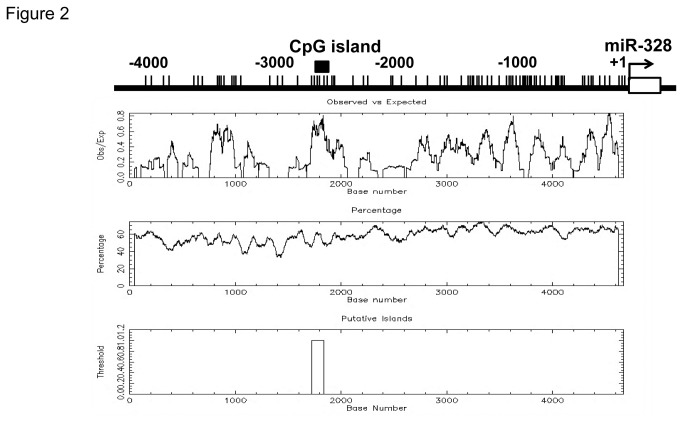
Surveyed CpG island in the miR-328 5’-flanking region. A single CpG island was predicted spanning bp -2459 to -2343 (criteria used: Island size > 100 bp, GC percent > 50.0%, ratio of observed (Obs) CpG sites to expected (Exp) CpG sites > 0.6). The vertical lines on the top horizontal line indicate the cytosine resides of CpGs and the black box indicates the identified CpG island. Numbers in the top panel represent nucleotide positions from miR-328. Second panel, distribution of observed/expected ratios of CpG dinucleotides; third panel, distribution of C+G; lower panel, identification of the putative CpG island within the -4.1 kb of analyzed sequence. The reference sequence was derived from NCBI sequence database (NC_000016.9, complement).

We focused on DNA methylation in the miR-328 5’-flanking region. CpG islands were identified using CpGplot software [44]. We detected one CpG island in the miR-328 5’-flanking region from bp -2459 to -2343 (**Figure 2**).

### Up-regulation of miR-328 expression by treatment with a DNA demethylating agent in BCRP-expressing cell lines

**Figure 3:**
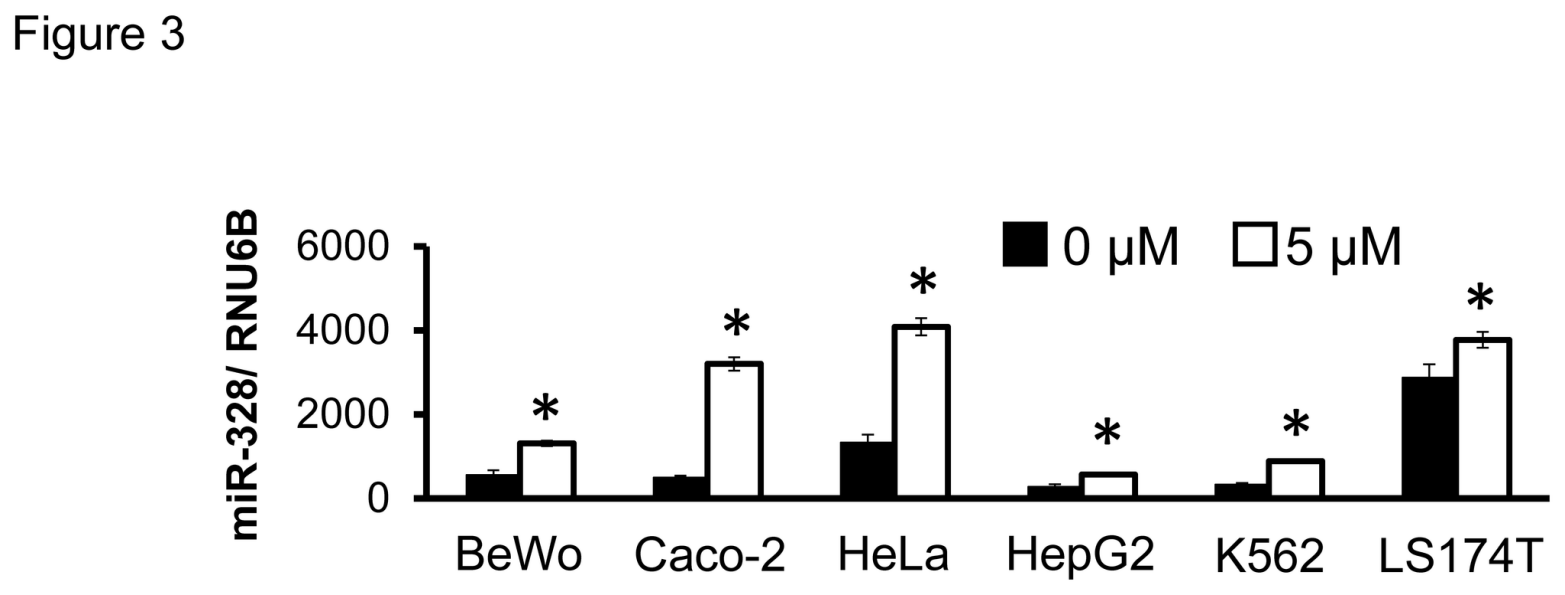
Effect of demethylation on miR-328 levels in BCRP-expressing cell lines. Results represent the mean ± SD for three independent experiments. *, *P* < 0.05.

To select BCRP-expressing cells, BCRP mRNA was measured. LS174T, HeLa, Caco-2, BeWo, HepG2 and K562 cells were treated with 5 µM 5-aza-2’-deoxycytidine (5-aza-dC, an inhibitor of DNA methylation) for 72 hours. All cell lines showed significantly increased miR-328 levels after the treatment (**Figure 3**).

### Transcriptional activity in the differentially methylated miR-328 5’-flanking region

**Figure 4:**
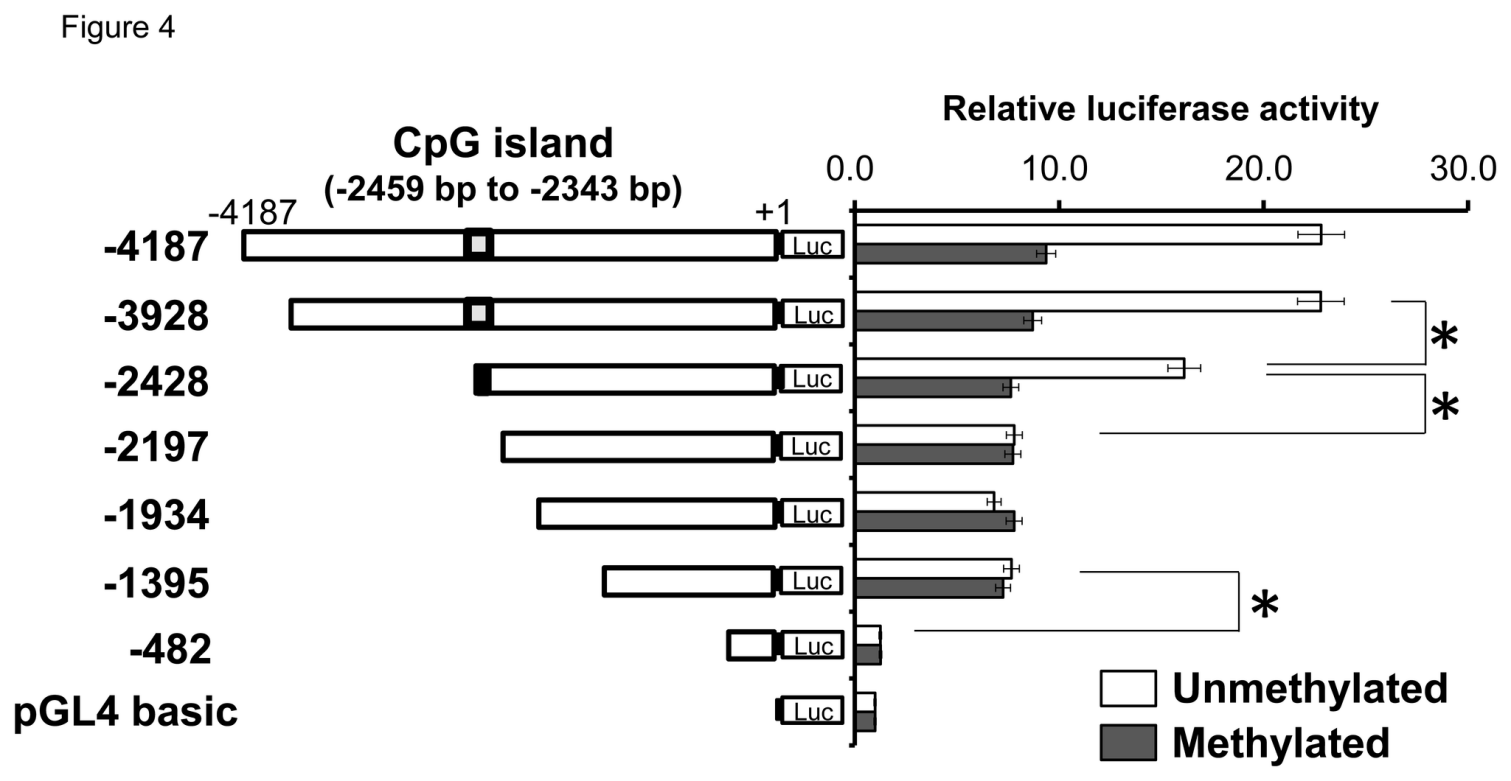
Transcription profiles of some deleted sequences under unmethylated/methylated conditions in the miR-328 5’-flanking region. Luciferase activity of unmethylated reporter constructs (opened bars) compared with the methylated constructs (filled bars) in BeWo cells. Results represent the mean ± SD for three independent experiments. Data are expressed as fold-increase in pGL4.10 activity, which was assigned a value of 1.00. *P < 0.05 vs. methylated control.

To identify important regions and to evaluate the effect of methylation on the transcriptional activity, luciferase assays using methylated and unmethylated constructs containing various segments of the miR-328 5’-flanking region were conducted (**Figure 4**). Activity levels were markedly higher in unmethylated constructs containing the regions (−4187 to +1), (−3928 to +1) and (-2428 to +1) than in methylated constructs. The construct with (-1395 to +1) showed significantly higher activity than that with (-482 to +1) in both a methylated and unmethylated state, suggesting that the region (-1395 to -482) was important for regulating the expression independent of DNA methylation. The activity in the unmethylated vector with (-3928 to +1) was significantly higher than with (-2428 to +1) (*P* < 0.05), and the unmethylated vector containing (-2428 to +1) showed significantly higher activity than that with (-2197 to +1) (*P* < 0.05). These results suggest that methylation between positions -3928 and -2197 was important for transcriptional activity.

### Inhibition of C/EBPα expression down-regulates miR-328 expression

**Figure 5:**
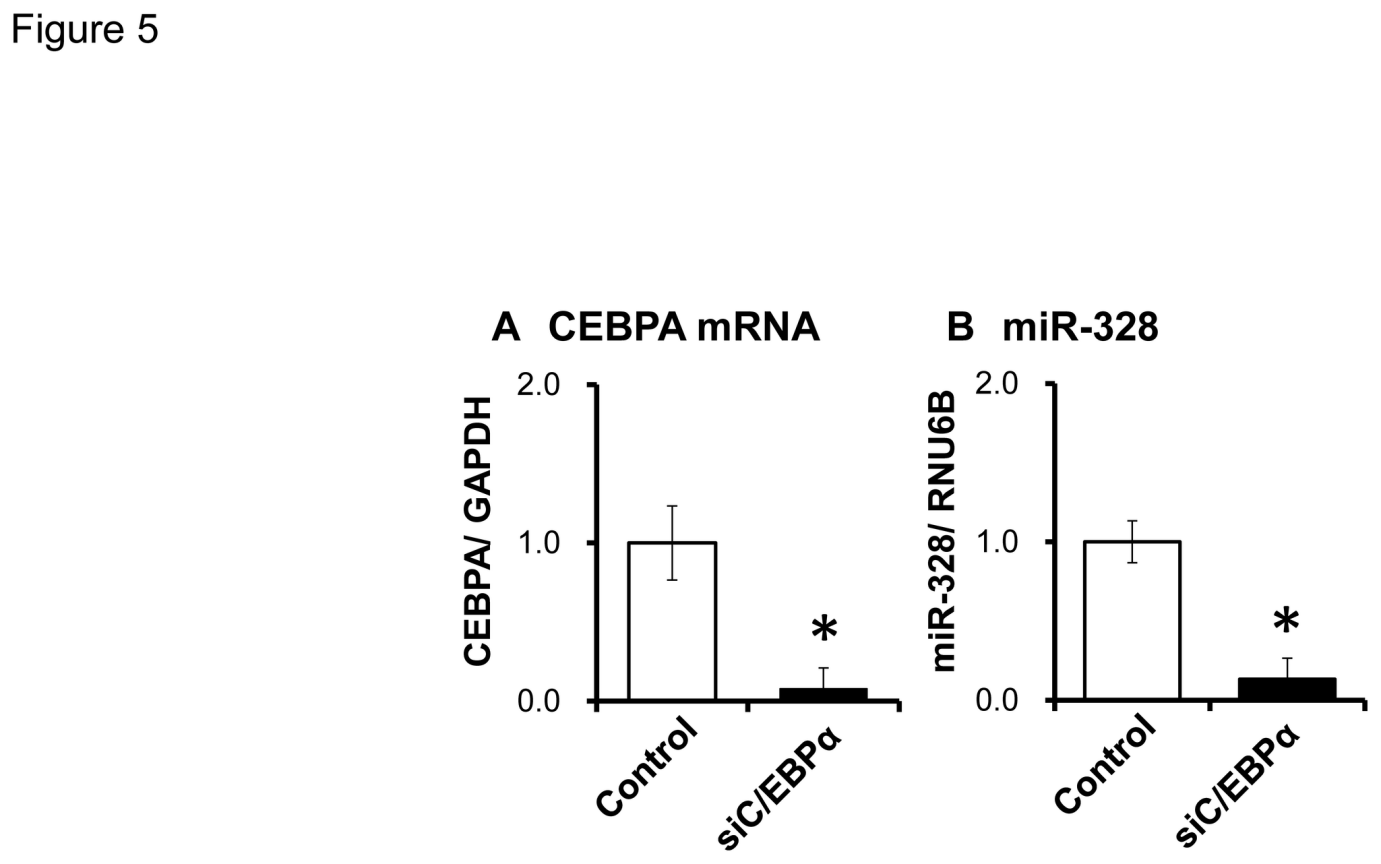
Effect of C/EBPα knockdown on C/EBPα and miR-328 levels in BeWo cells. The data are shown relative to negative control #1 siRNA-transfected cells. Results represent the mean ± SD for three independent experiments. *, *P* < 0.05.

Transcription factors capable of binding to the sequence from bp -3928 to -2197 of the miR-328 5’-flanking region were screened using TFSEARCH [45], TRANSFAC ver. 8.3 [46,47] and TFBIND [48]. CCAAT/enhancer binding protein α (C/EBPα) was selected by all three programs. To confirm the involvement of C/EBPα in miR-328 expression, C/EBPα siRNA was transfected into BeWo cells. The C/EBPα mRNA level was completely reduced after transfection (**Figure 5A**). C/EBPα siRNA also significantly diminished miR-328 levels (**Figure 5B**).

### Methylation-dependent C/EBPα-binding to the miR-328 5’-flanking region

**Figure 6:**
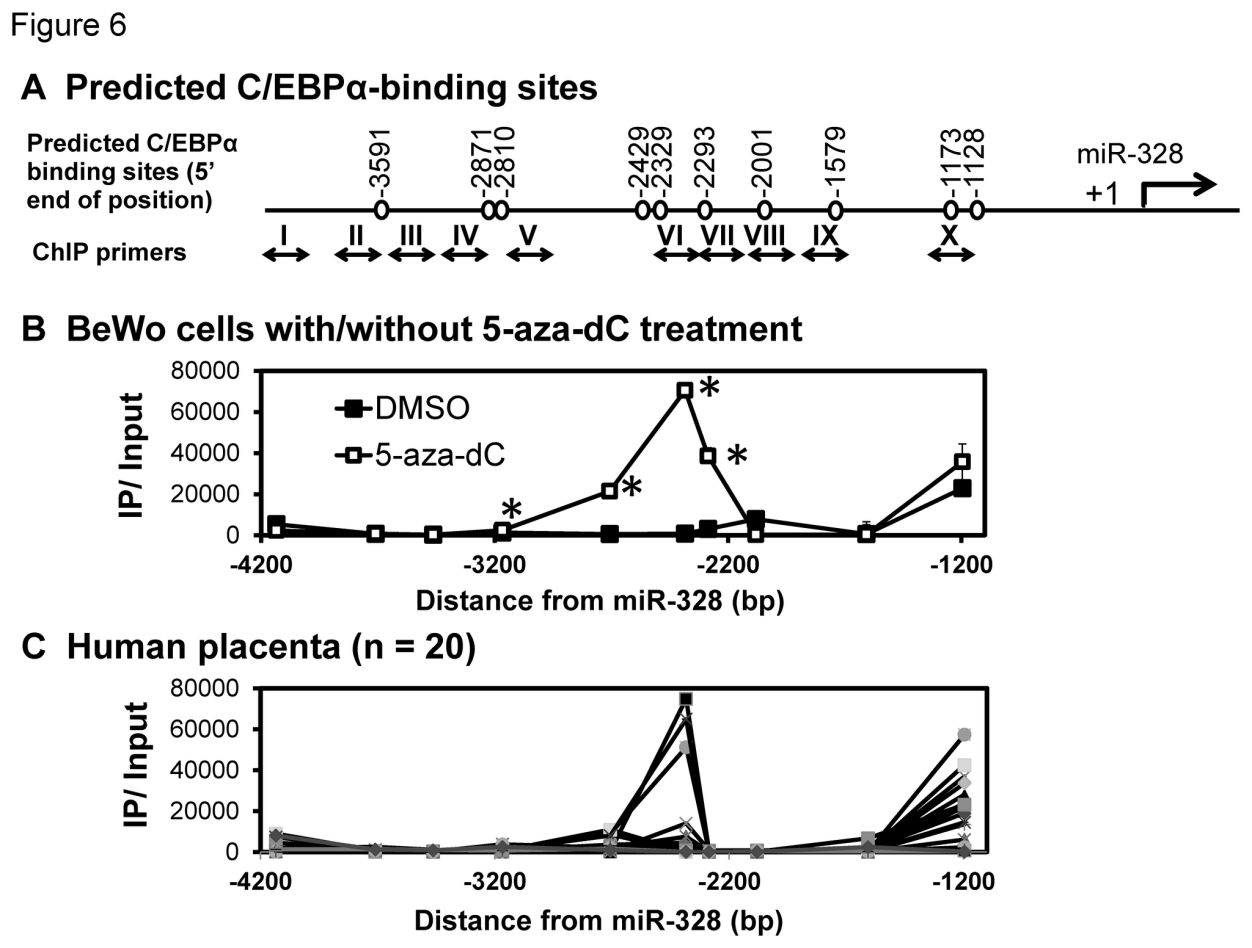
C/EBPα-binding frequency in the miR-328 5’-flanking region. (A) Schematic representation of predicted binding sites of C/EBPα in the miR-328 5’ flanking region. The position numbers indicate the 5’ end of the C/EBPα motif. Primers for the ChIP assay are also indicated. (B) Comparison of C/EBPα-binding frequency in the miR-328 5’ flanking region. Open and closed squares indicate the results of 5-aza-dC and DMSO (control) treatment, respectively, in BeWo cells. Immunoprecipitated DNA was amplified with ten specific primers (I-X described as double-headed lines). (C) C/EBPα-binding frequency in human placentas (n = 20). Input, DNA isolated from the lysate before immunoprecipitation; IP, DNA immunoprecipitated with anti-C/EBPα antibody. Data are expressed as the mean ± SD for three independent experiments.

To investigate the association between C/EBPα-binding frequency and methylation patterns in the miR-328 5’-flanking region, a chromatin immunoprecipitation (ChIP) analysis was performed. **Figure 6A** shows the C/EBPα-binding sites predicted using Transfac ver. 8.3. All the predicted sites were proximal to the CpG dinucleotide. C/EBPα-binding frequency was analyzed in the miR-328 5’-flanking region in BeWo cells using 5-aza-dC. For real-time PCR, ten primer sets (ChIP primers I to X, see **Figure 6A**) that amplified 5’-flanking regions including predicted C/EBPα-binding sites were designed. After treatment with 5-aza-dC, the C/EBPα-binding frequency was increased in four regions (ChIP primers IV, V, VI and VII) compared to DMSO-treated cells as a negative control (**Figure 6B**). C/EBPα-binding was also detected in ChIP primer X amplifying the region from -1196 to -1096 bp, but independent of the methylation. When we tested the C/EBPα-binding frequency in human placental samples, large inter-individual variability was observed in ChIP primers VI and X (n = 20) (**Figure 6C**).

### Promoter deletion analysis in the miR-328 5’-flanking region

**Figure 7:**
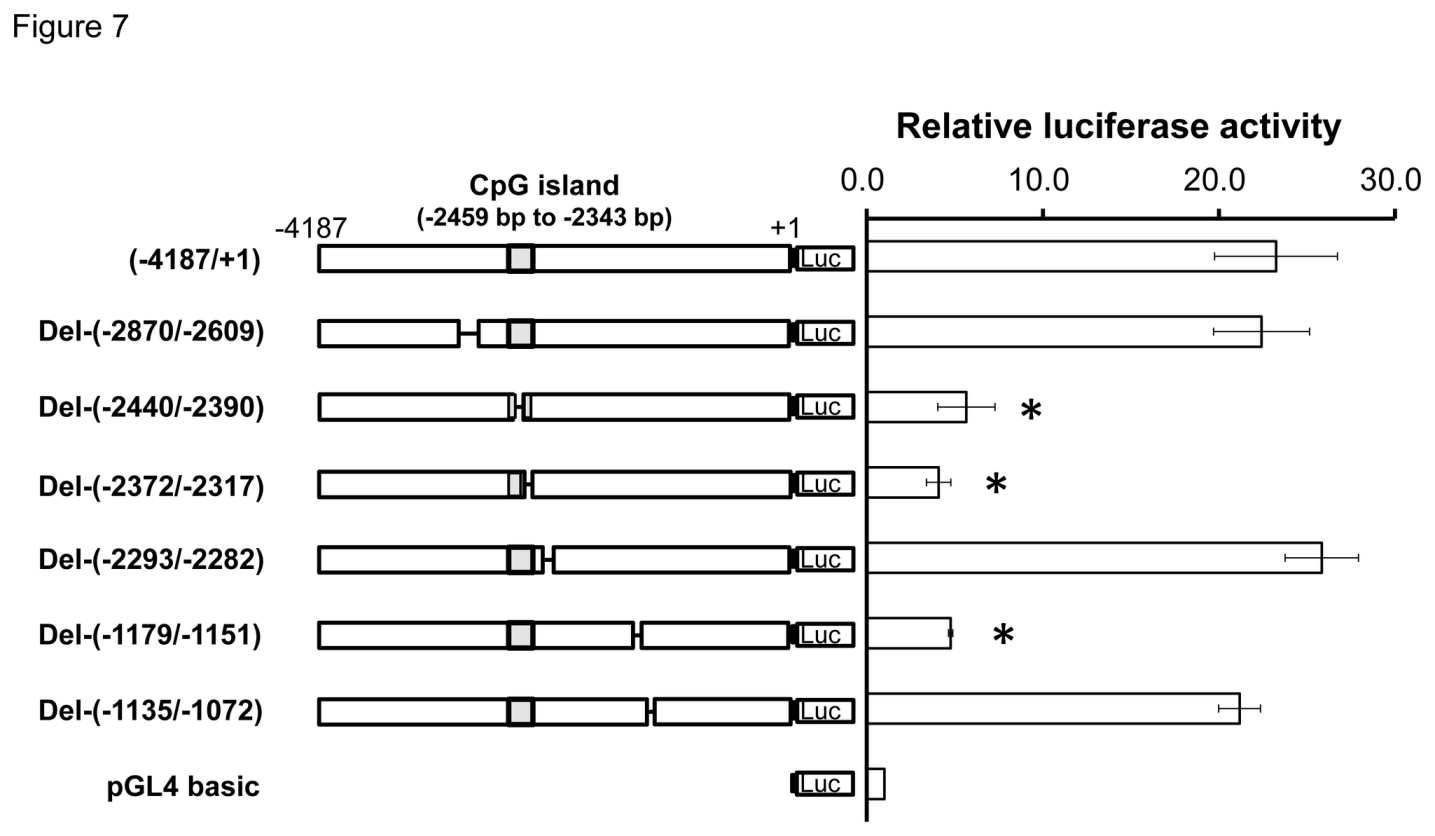
Transcription profiles of deleted sequences of predicted C/EBPα-binding sites in the miR-328 5’-flanking region. Positions of deleted regions are indicated with ‘Del-’. Values are the mean ± SD for three independent experiments. *, *P* < 0.05 compared with the full-length construct.

To identify the functionally important C/EBPα-binding sites, we constructed a series of deletion plasmids for seven C/EBPα-binding sites (position -2871, -2810, -2429, -2329, -2293, -1173, and -1128, **Figure 6A**), and evaluated their transcriptional activity using luciferase assays. Deletion of (-2870 and -2609), (-2293 and -2282), and (-1135 and -1072) had no effect on the transcriptional activity. However, constructs lacking (-2440 to -2390), (-2372 to 2317) and (-1179 to -1151) showed extremely low transcriptional activity compared with the full-length vector (**Figure 7**).

### Confirmation of C/EBPα-binding to predicted motif sequences

**Figure 8:**
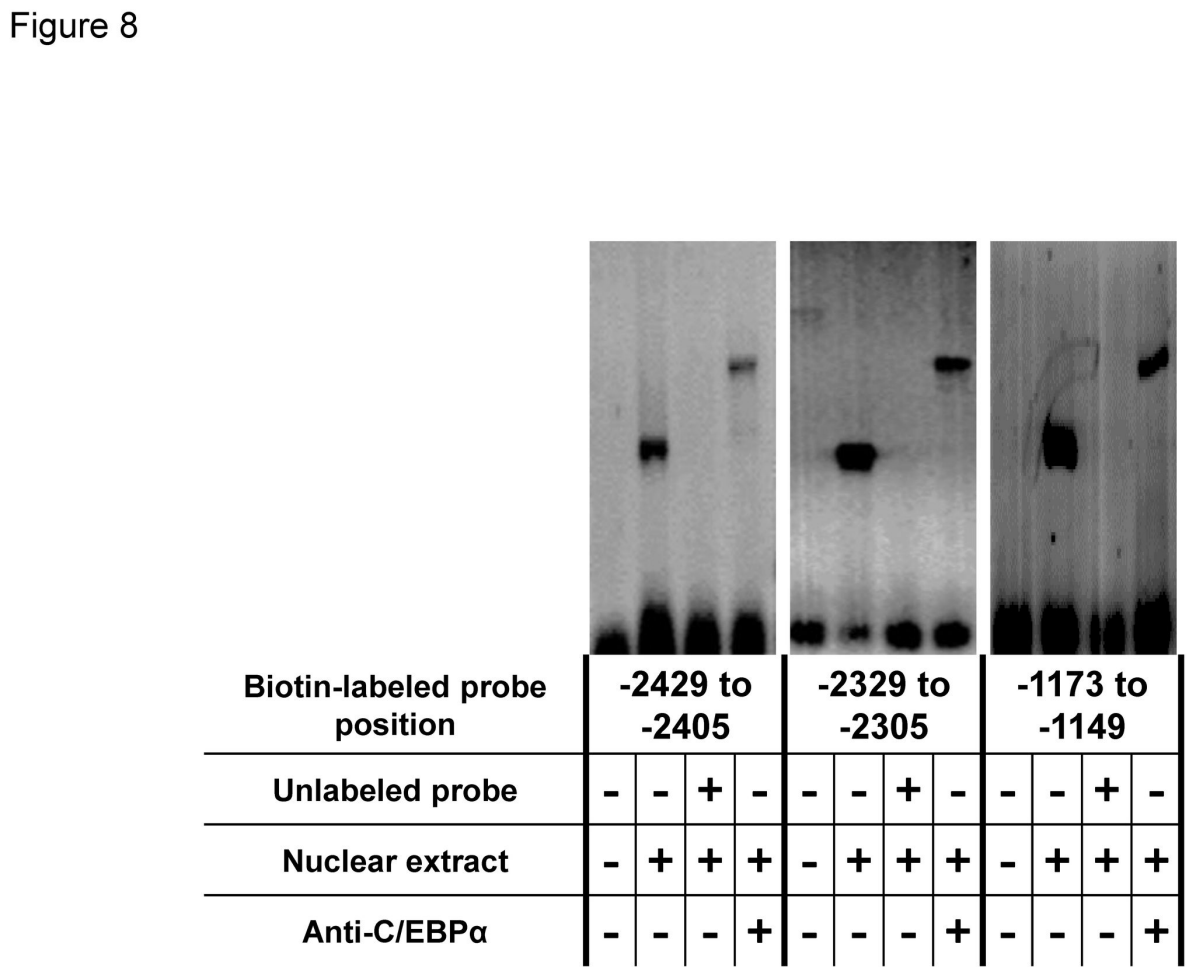
Confirmation of C/EBPα-binding sites in the miR-328 5’-flanking region.

A gel mobility shift assay (EMSA) was performed to confirm that C/EBPα binds to the predicted motif sequences. We used biotin-labeled double-stranded DNA corresponding to the C/EBPα-binding sequences. The results showed that all three probes formed complexes with nuclear proteins from BeWo nuclear extracts (**Figure 8**). The specificity of these protein-DNA complexes was confirmed in competition assays using each unlabeled oligonucleotide. Anti-C/EBPα antibodies led to the formation of supershifted complexes, indicating that C/EBPα binds to the predicted motifs.

### Correlation between C/EBPα-binding frequency and miR-328 levels in the human placenta

**Figure 9:**
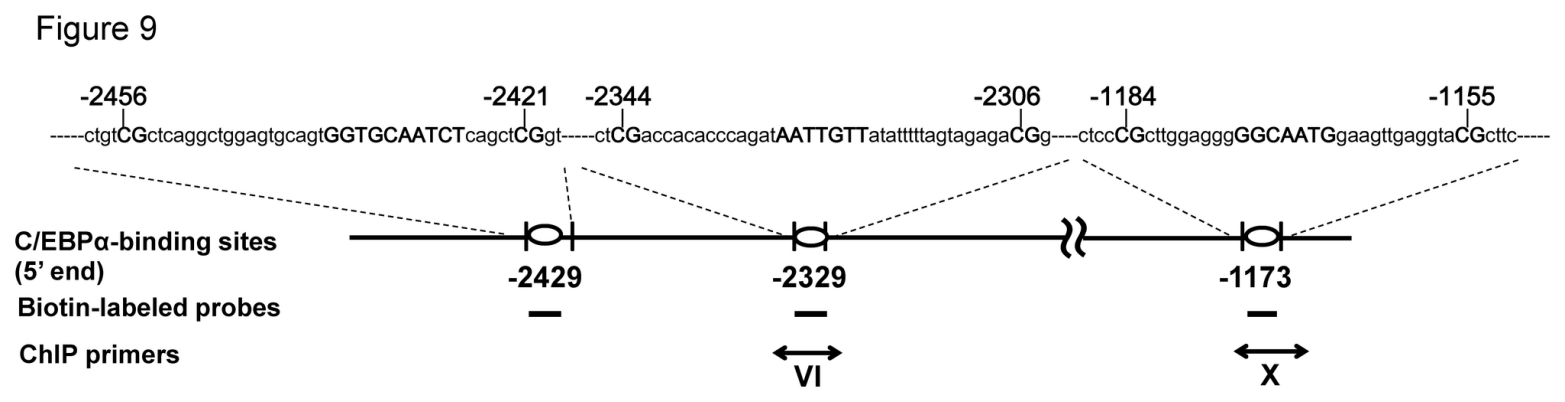
Schematic representation of identified C/EBPα-binding sites in the miR-328 5’-flanking region. Putative C/EBPα-binding sites and CpG dinucleotides are indicated in bold capital letters. All numbers indicate the relative distance from the 5’ end of the miR-328 transcript. Each vertical bar represents the CpG dinucleotide closest to the C/EBPα-binding site (circle). The three probes for EMSA and two primers (VI and X) for ChIP assays are indicated by horizontal lines and double-headed lines, respectively.

**Figure 10:**
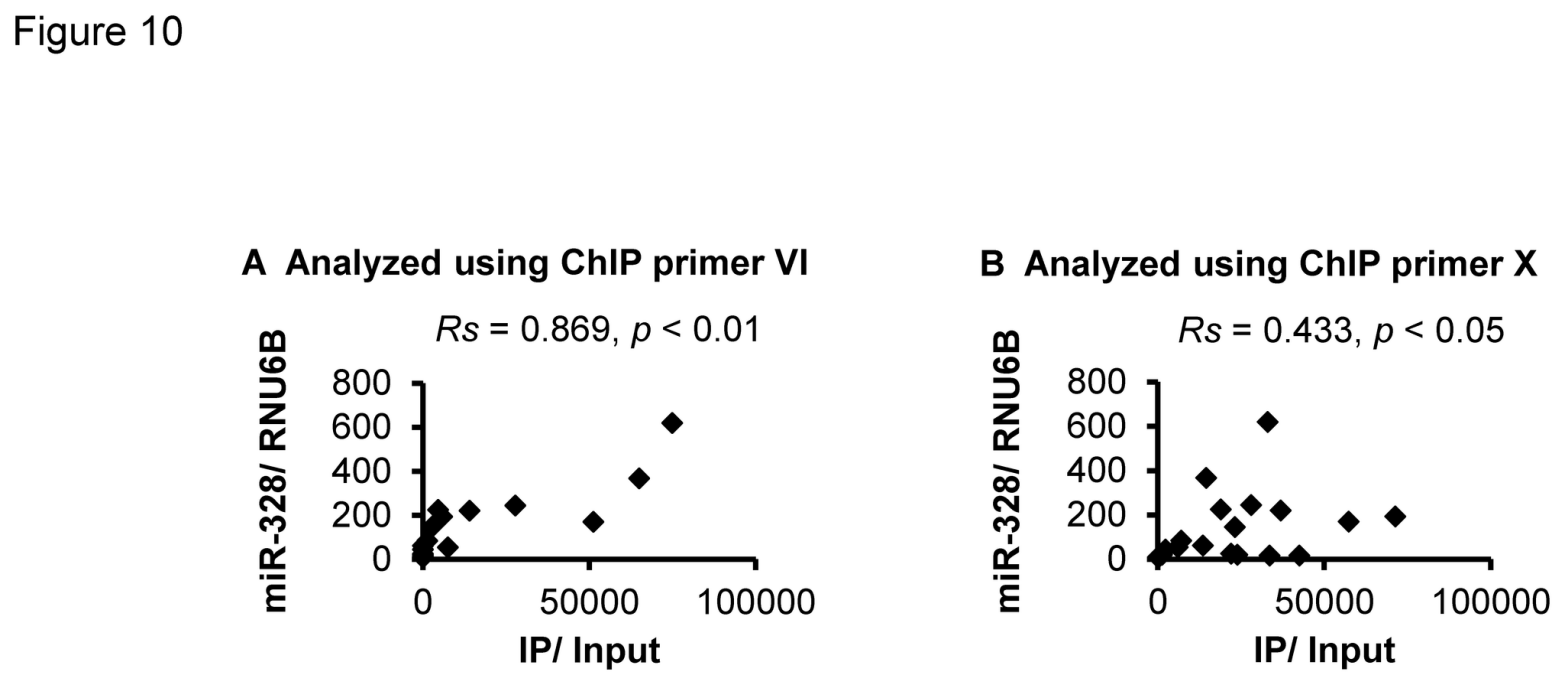
Correlation between C/EBPα-binding frequency and miR-328 levels in placental samples (n =20). (A) and (B) show C/EBPα-binding frequency obtained with ChIP primer VI and X, respectively.

We analyzed the association between binding frequencies in the regions around the three C/EBPα-binding sites (**Figure 9**) and miR-328 levels in the human placenta (n = 20). Binding frequencies by ChIP primer VI (-2429 and -2329) and primer X (-1173) were significantly correlated with miR-328 levels (**Figure 10**). Notably, a significantly higher correlation was observed in the region analyzed using ChIP primer VI. These results suggest that the C/EBPα-binding frequency is a determinant of human placental miR-328 levels.

### Influence of methylation patterns and C/EBPα levels on C/EBPα-binding frequency in the human placenta

**Figure 11:**
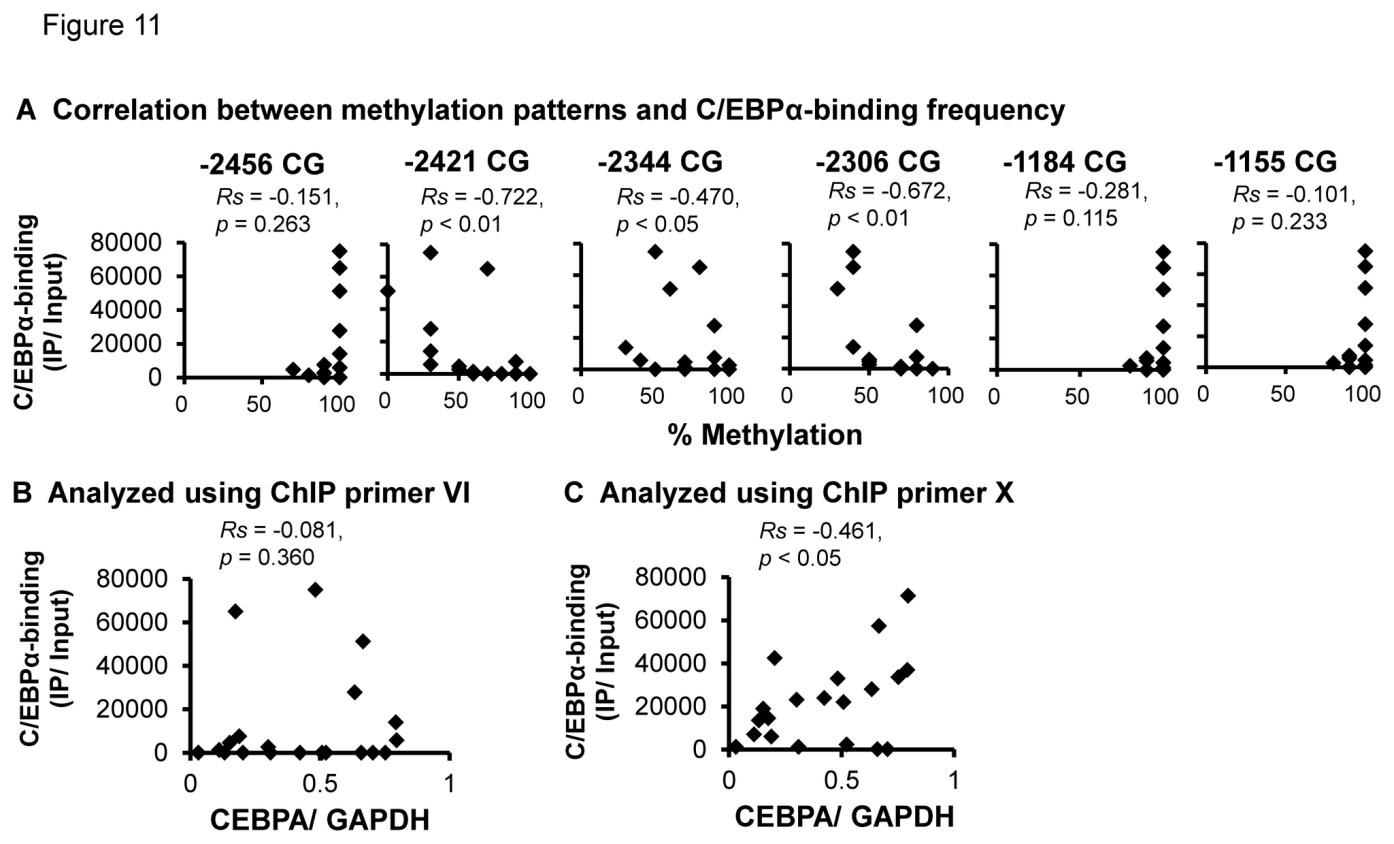
Effect of methylation patterns (A) and C/EBPα levels (B and C) on C/EBPα-binding in placental samples (n = 20). The C/EBPα-binding frequency of four CpG dinucleotides (-2456CG, -2421CG, -2344CG, -2306CG) was obtained from ChIP primer VI. The other two (1184CG, -1155CG) were from primer X.

The association between the methylation levels in the CpG dinucleotides proximal to the C/EBPα-binding sites and C/EBPα levels was evaluated. We focused on the six CpG dinucleotides (-2456CG, -2421CG, -2344CG, -2306CG, -1184CG and -1155CG) close to identified C/EBPα-binding sites (**Figure 9**). Methylation patterns at three CpG dinucleotides (-2421CG, -2344CG and -2306CG) were negatively correlated with C/EBPα-binding frequency (**Figure 11A**). The role of C/EBPα levels in C/EBPα-binding was also evaluated. No correlation was found in the region amplified by ChIP primer VI (**Figure 11B**), but a significant correlation was observed using primer X (**Figure 11C**).

### Influence of methylation patterns and C/EBPα levels on miR-328 levels in the human placenta

**Figure 12:**
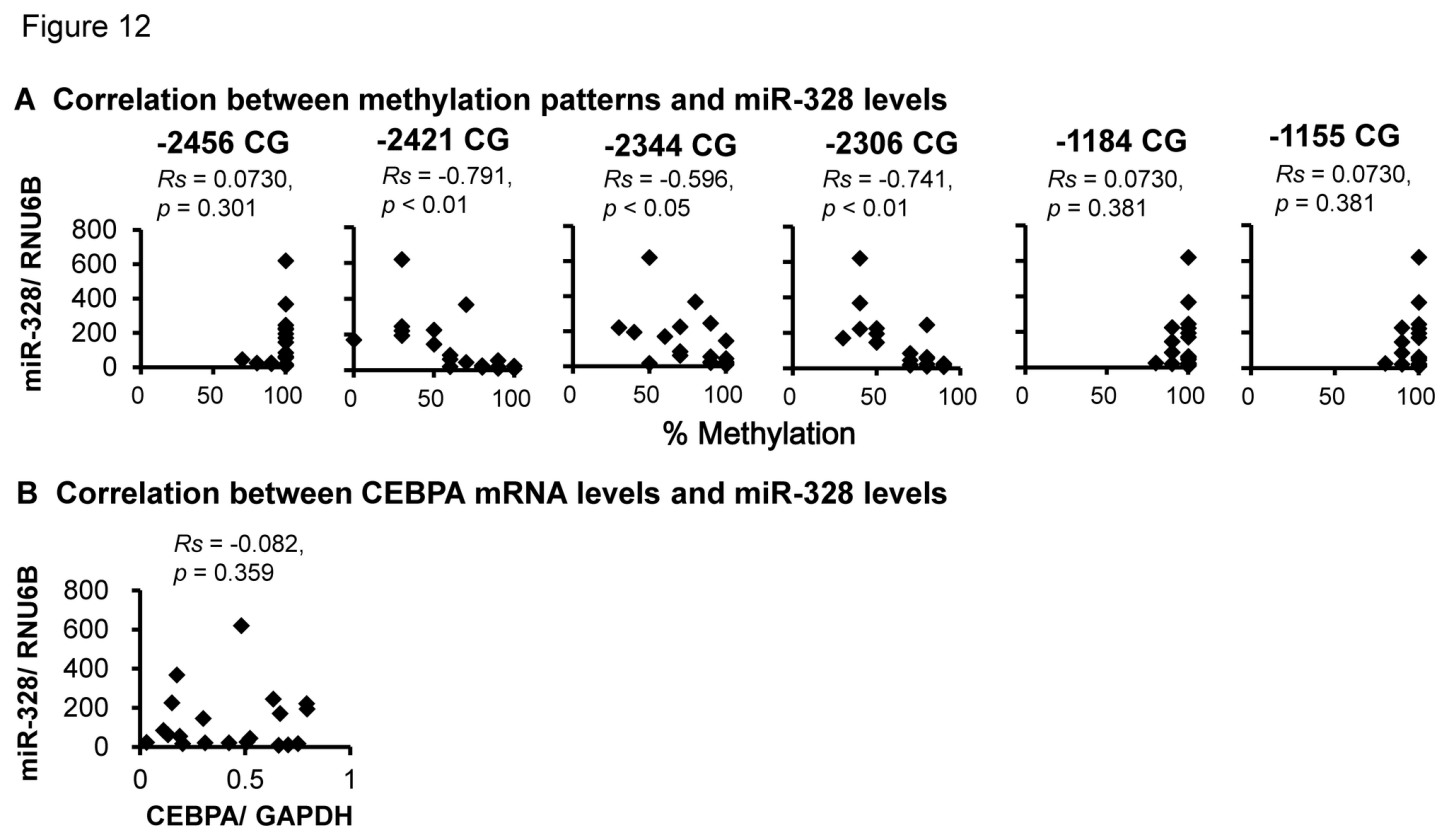
Effect of methylation patterns (A) and C/EBPα levels (B) on miR-328 levels in placental samples (n = 20).

Methylation patterns at the three CpG dinucleotides (-2421CG, -2344CG and -2306CG) were also negatively correlated with miR-328 levels (**Figure 12A**). C/EBPα levels showed no correlation with miR-328 levels, indicating they were not crucial to the individual differences in miR-328 levels in the human placenta (**Figure 12B**).

### Relationship between methylation patterns and placental BCRP mRNA and protein levels

**Figure 13:**
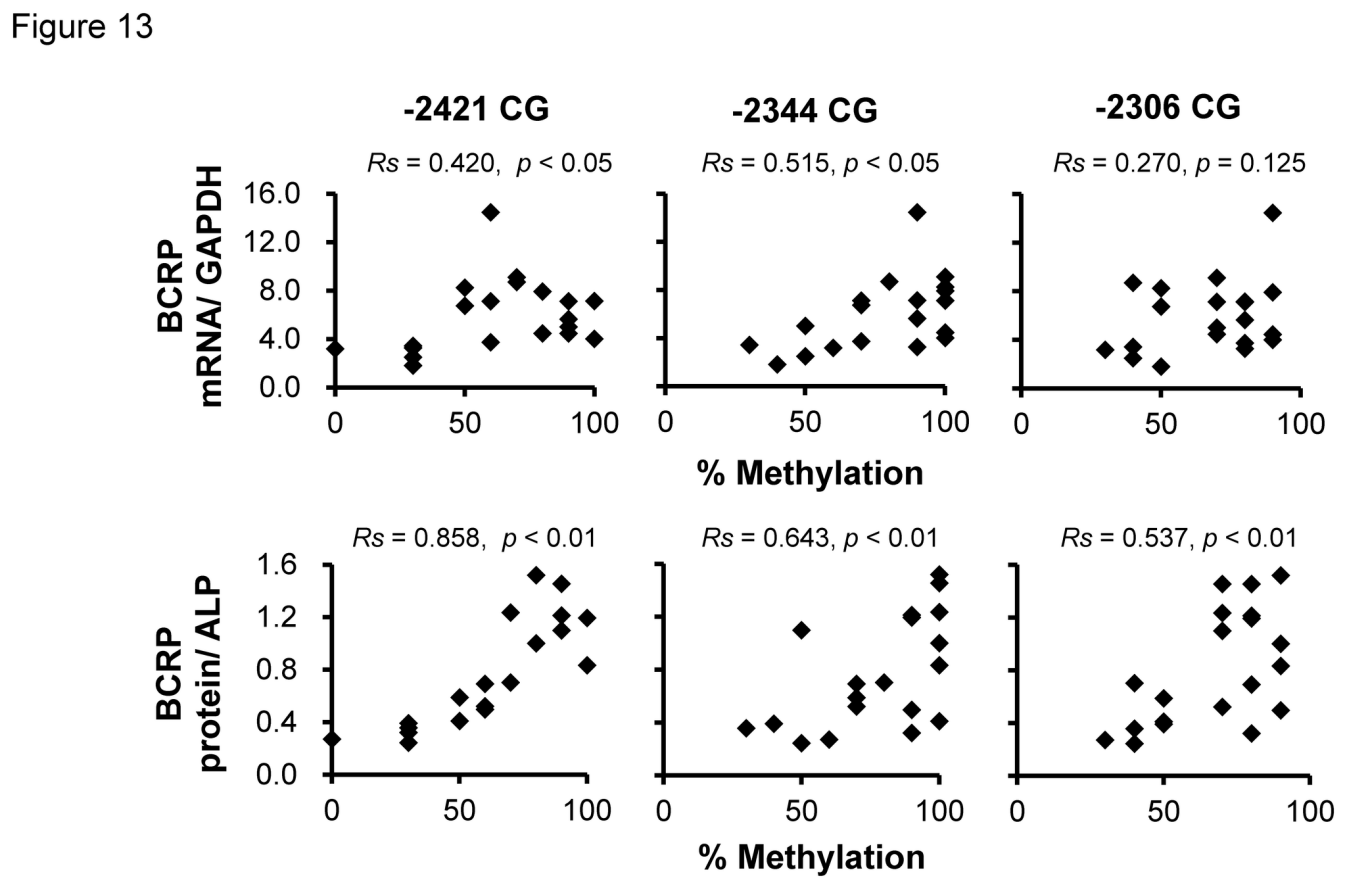
BCRP mRNA (upper) and protein (lower) levels in the human placenta and methylation patterns in the miR-328 5’-flanking region.

The association between the methylation patterns of the three CpG dinucleotides (-2421CG, -2344CG and -2306CG) which correlated with C/EBPα-binding frequency and miR-328 levels, and BCRP levels was analyzed. The methylation level of -2421CG and -2344CG but not -2306CG positively correlated with BCRP mRNA levels (**Figure 13**). With regard to the protein levels, significantly positive correlations were observed at all three CpG dinucleotides. These results suggest that methylated CpG dinucleotides in the miR-328 5’-flanking region suppressed miR-328 expression, leading to BCRP up-regulation in the human placenta.

## Discussion

Human placenta shows the highest BCRP expression of all tissues [49]. Thus, much effort has been made to describe the pharmacological roles of BCRP at the maternal-fetal interface [49,50]. BCRP is located mainly on the apical membrane in syncytiotrophoblasts where it pumps substrates into the maternal circulation. In mice, Bcrp was shown to limit fetal exposure to substrate drugs [51–54]. In human placental trophoblasts, BCRP gene polymorphisms play a limited role in the inter-individual variability of BCRP levels [3]. In this study, to explore the mechanisms behind the variability in placental BCRP levels, we focused on the association between miR-328 and BCRP levels. As shown in **Figure 1**, miR-328 levels were significantly correlated with BCRP mRNA and protein levels. These observations are consistent with a previous report showing BCRP mRNA degradation by miR-328 [20,55].

Although it is now quite clear that BCRP plays an important role in protecting the fetus against potential toxicity of drugs and xenobiotics, few studies have been done to demonstrate the physiological role of BCRP in the placenta. Evseenko et al. showed that inhibition of BCRP activity by a BCRP-specific inhibitor Ko143 increased cytokine-induced apoptosis in primary trophoblasts and BeWo cells, and BCRP expression in placenta from pregnancies with idiopathic fetal growth restriction was significantly lower than that from normal pregnancies [56,57]. These authors proposed that BCRP may play an unrecognized role in the placenta, protecting trophoblasts against apoptosis induced by cytokines, and BCRP is likely a survival factor in differentiation of placental trophoblasts. In human placenta, the role of DNA methylation has been studied extensively. Several recent studies have examined the association between placental methylation change and neonatal outcome [58], and showed that placental miRNA expression profiles were associated with fetal growth [59]. Decreased BCRP expression associated with high miR-328 expression resulting from the demethylation in 5’-flanking region may result in placental function deficit, thus contributing to fetal growth restriction. However, analyzed placental samples in this study had no information about the demographics, because the purpose of this study focused on the association analysis between BCRP and miR-328 expression. Further studies are needed to confirm this hypothesis.

A single CpG island was predicted in the miR-328 5’-flanking region. Since methylation in CpG islands is known to regulate gene expression, we analyzed methylation patterns of CpG dinucleotides in the island. Before the luciferase analysis, the effect of demethylation on miR-328 expression was tested using BCRP-expressing cells. In luciferase reporter assays in BeWo cells, we found that the region (-1395 to -482) regulates the expression, independent of DNA methylation. On the other hand, methylation in (-3928 to -2197), the region containing the CpG island, strongly suppressed the miR-328 expression. Next, we selected C/EBPα as a candidate transcription factor which binds to the CpG island, and confirmed its involvement in miR-328 expression using C/EBPα siRNA-transfected BeWo cells (**Figure 5**). C/EBPα knock-down was associated with an extremely low expression of miR-328, suggesting that C/EBPα is the major activator for miR-328 expression. To investigate the role of methylation in the binding of C/EBPα to the miR-328 5’-flanking region, the C/EBPα-binding frequency was determined in BeWo cells. Treatment of BeWo cells with 5-aza-dC increased the C/EBPα-binding frequency in four regions (ChIP primers IV, V, VI and VII) in the miR-328 5’-flanking region (**Figure 6**). The region with relatively high binding frequency under demethylation was approximately -3167 to -2184 (see **Table S1**). Interestingly, this region was close to (-3928 to -2197) showing significantly higher luciferase activity in unmethylated constructs (**Figure 4**). In human placental samples, the C/EBPα-binding frequency varied inter-individually in this region (**Figure 6C**). These results suggest that methylation from bp -3167 to 2184 in the miR-328 5’-flanking region is important for C/EBPα-mediated miR-328 expression.

By deletion analysis (**Figure 7**) and EMSA (**Figure 8**), positions -2429, -2329 and -1173 bp within the region showing relatively high C/EBPα-binding frequencies in BeWo cells were identified as binding sites for C/EBPα. In human placenta, a strong association between C/EBPα-binding frequencies and miR-328 levels was also observed for DNA fragments (including the C/EBPα-binding sites at -2429 and -2329) obtained using ChIP primer VI (**Figure 10A**, *Rs* = 0.869, *P* = 4.89E-07).

Among six CpG dinucleotides (-2456, -2421, -2344, -2306, -1184, and -1155) within the C/EBPα-binding sites, the methylation levels of the three CpG dinucleotides (-2421CG, -2344CG and -2306CG) proximal to the two C/EBPα-binding sites (-2429 and -2329 bp) were negatively correlated with C/EBPα-binding frequencies and miR-328 levels, but not for -1184CG and -1155CG dinucleotides proximal to the C/EBPα-binding site (-1173 bp). The absence of an association for the -1184CG and -1155CG dinucleotides was consistent with the results of luciferase assays suggesting that miR-328 expression is regulated by C/EBPα independently of DNA methylation. Since the C/EBPα-binding frequency for DNA fragments (including the C/EBPα-binding site at -1173 bp) obtained with ChIP primer X was significantly correlated with C/EBPα levels, there are two factors, methylation patterns and C/EBPα levels, behind the C/EBPα-binding frequency. However, as shown in **Figure 12B**, miR-328 levels were not directly affected by C/EBPα levels.

Therefore, the DNA methylation pattern of three CpG dinucleotides (-2421CG, -2344CG and -2306CG) plays a major role in regulating the binding of C/EBPα to the miR-328 5’-flanking region, leading to inter-individual differences in miR-328 levels in the human placenta.

Finally, we showed that methylation levels at the three CpG dinucleotides (-2421CG, -2344CG and -2306CG) were significantly correlated with BCRP protein levels in human placentas (**Figure 13**). Methylation status in the human placenta is profoundly affected by environmental factors and drug exposure, due to its role at the interface between the maternal and fetal circulations [60–63]. Interestingly, it was reported that drug-induced hyper-methylation involves resistance to azidothymidine [64], etoposide, nalidixic acid, doxorubicin, vinblastine, colchicine, cisplatin, 5-fluorouracil, 5-fluorodeoxyuridine, and methotrexate in human cancer cell lines [65]. DNA methylation in the miR-328 5’-flanking region is suggested to be associated with an increase of BCRP expression, leading to drug resistance in cancers. In this study, analyzed placental samples were relatively small size (n = 20). Additional study using larger sample size is needed to determine the role of DNA methylation in the inter-individual differences in miR-328 and BCRP expression. However, our results are important first step to investigate how DNA methylation in the miR-328 5’-flanking region, and to identify the association between miR-328 and BCRP expression levels in human placenta.

## Materials and Methods

### Tissue samples

Twenty human placental samples were obtained from patients who underwent a normal pregnancy at Tottori University Hospital. All placental samples were excised from each individual placenta. Placenta tissues were macroscopically separated from decidua. Tissues were frozen in liquid nitrogen immediately after delivery and stored at -80°C.

### Ethics Statement

Each patient gave written informed consent for her sample to be used for scientific research, which was approved by the Tottori University Ethics Committee.

### Genotyping of BCRP

The procedure used to genotype BCRP G34A, C376T, and C421A mutations was described previously [3].

### RNA Extraction and cDNA Synthesis

Total RNA extraction and RT-PCR procedures for placental samples were described elsewhere [66]. RT was performed in a 20-µL reaction mixture containing 5 µg of total RNA in 1×First-strand Buffer, 25 mM DTT, 0.5 µg of the random primers (Promega, Madison, WI), 2 mM of each deoxynucleoside-5’-triphosphate, and SuperScript II RNase H- reverse transcriptase (Invitrogen, Carlsbad, CA). Samples were incubated at 42°C for 1 h. As a negative control, template RNA was processed without the reverse transcriptase.

### Quantification of BCRP mRNA and protein levels

The quantitative real-time PCR for BCRP mRNA levels and western blotting for BCRP protein levels were performed as described previously [3].

### TaqMan-based real-time quantification of miRNA

The expression of mature miR-328 was quantified by quantitative real-time PCR using TaqMan miRNA assays according to the manufacturer’s protocols (Applied Biosystems, Foster City, CA). U6 small nuclear RNA (RNU6B; Applied Biosystems) served as an internal normalized reference. The relative quantity for miR-328 was determined by the comparative Ct method (2 –*ΔΔ*Ct).

### Cell culture

The LS174T, Caco-2, BeWo, HepG2 and K562 cell lines were obtained from Dainippon Pharmaceuticals (Osaka, Japan). The HeLa cell line was obtained from the RIKEN, Cell Bank (Tsukuba, Japan). All cell lines were grown at 37°C in an incubator with 5% CO_2_. The LS174T cell line was maintained in Eagle’s minimum essential medium. The Caco-2, HeLa and HepG2 cell lines were maintained in Dulbecco’s modified Eagle’s medium. The BeWo cell line was maintained in Ham’s F12 Kaighn’s modification medium. The K562 cell line was maintained in RPMI 1640 supplemented with 2.5 mM glutamine (Life Technologies, Carlsbad, CA). All media were supplemented with 10% fetal bovine serum.

### Demethylation experiments

To block DNA methylation, all cells were treated with 5 µM 5-aza-2’-deoxycytidine (5-aza-dC; Sigma Aldrich, St Louis, MO) and DMSO (0.01%, final concentration) as vehicle control for 72 h. The cells were dissociated enzymatically using TrypLE Express (Invitrogen), washed, and resuspended in phosphate-buffered saline (PBS). Small RNA was immediately extracted from the collected cells using the mirVana isolation Kit (Ambion, Austin, TX) according to the manufacturer’s protocol.

### DNA extraction and bisulfite sequencing

Genomic DNA was extracted from 20 placental tissues and then subjected to sodium bisulfite treatment using an Epitect bisulfite modification kit (Qiagen, Valencia, CA). We amplified and sequenced the proximal 5’-flanking miR-328 for methylation analysis. These regions were amplified using two primer sets (primer 1 for bp -2510 to -2145 and primer 2 for bp -1287 to -918, see **Table S2**). The PCR product amplified with primer 1 was used for the nested second PCR (primer 1s). The initial denaturation was for 9 min at 95°C, followed by 45 cycles for 40 s at 98°C, 45 s at 54°C and 20 s at 72°C and a final elongation for 5 min at 72°C. The PCR products were separated by 3% agarose gel electrophoresis, extracted and then cloned into the pGEM-T easy vector (Promega). After bacterial amplification of the cloned PCR fragments by standard procedures, 10 clones were subjected to DNA sequencing.

### Construction of 5’-flanking reporter plasmids

To explore the transcriptional regulation of miR-328, we generated a longer fragment of the miR-328 5’-flanking region (-4230 to +36 relative to the transcription start site of miR-328) using two sets of primers (described as ins1 and ins2 in **Table S3**). pGL4-(-4230/+36) was used as a template to generate a series of vectors of various lengths. Each amplified fragment was digested and inserted into the pGL4.10 basic vector (Promega) between the *Kpn*I and *Xho*I sites (primer sets described in **Table S3**). These plasmids were then sequenced for confirmation.

### *In vitro* DNA methylation of luciferase reporter constructs

The series of vectors were methylated *in vitro* using the bacterial methylase *Sss*I (New England Biolabs, Ipswich, MA). To generate completely-methylated vectors, each vector was digested with *Kpn*I and *Xho*I to release the inserted sequence. Each fragment (4 µg) was incubated at 37°C for 4 h with 40 U of *Sss*I supplemented with 160 µM *S*-adenosylmethionine. After methylation, each fragment was religated with the pGL4.10 vector. The unmethylated insert sequence was extracted from *E. coli* JM110 (a dam and dcm DNA methylase-deficient strain).

### Cell transfection and luciferase assays

BeWo cells were transfected using Lipofectamine 2000 (Invitrogen) with 400 ng of each methylated or unmethylated vector. In each case, 100 ng of pRL-TK (Promega) was used to correct for the transfection efficiency. Luciferase activity was measured with the Dual-Luciferase Reporter Assay System (Promega). 5’-flanking activities were expressed as the ratio of *Firefly* luciferase to *Renilla* luciferase activity.

### Chromatin immunoprecipitation assay

Chromatin immunoprecipitation (ChIP) assays were performed using the immunoprecipitation kit (Roche Diagnostics, Germany) according to the manufacturer’s instructions. Briefly, BeWo cells were grown to 80% confluence in Ham’s F12 Kaighn’s modification medium supplemented with 10% fetal bovine serum. Cells were washed with PBS twice. DMSO (control) and 5-aza-dC-treated cells were fixed using 1% formaldehyde on ice for 5 min. Cells were incubated for 10 min in a 2.5 M glycine solution. After being re-suspended in SDS lysis buffer, the cells were sonicated until crosslinked chromatin was sheared to an average DNA fragment length of 200–500 bp. The sonicated lysate (200 µL) was used to quantify the total amount of DNA before immunoprecipitation (inputs). Immunoprecipitation was performed for 12-16 h at 4°C with either 1 µg of anti-C/EBPα antibody (Santa Cruz Biotechnology, Santa Cruz, CA) or a non-specific antibody (IgG). Precipitates were then washed three times with 1 mL of TE buffer and extracted with a DNeasy Mini kit (Qiagen). The average cycle threshold (Ct) was analyzed according to the 2(−*ΔΔ*Ct) method. The resulting data were divided by their corresponding input DNA. Fold enrichment was calculated with the ratio from the IgG control as a negative control to adjust the amplification efficiency of each primer. The primers and amplified regions are shown in **Table S1**.

### siRNA knock-down assay

Cells were plated in a 24-well plate at a density of 1.0× 10^5^ cells/mL/well. The transfection mix was prepared in Opti-MEM with siRNA and lipofectamine 2000 according to the manufacturer’s directions. The final concentrations of siRNA and lipofectamine added to the cells were 20-50 nM and 2 µL/ml, respectively. Cells were cultured in the presence of the transfection mixture for 48 h. After siRNA transfection, cells were harvested to analyze target genes expression levels and miR-328 levels by real-time PCR. The primers and amplified regions are shown in **Table S4**.

### Nuclear protein extraction

Nuclear extracts were prepared from cells using the NE-PER Nuclear and Cytoplasmic Extraction Reagents (Pierce Biotechnology, Rockford, IL). The protein concentration of each extract was measured at OD 595 nm. Each nuclear extract (5 µg) was subjected to an electrophoretic mobility shift assay.

### Electrophoretic mobility shift assay (EMSA)

3’ end biotin-labeled double-stranded oligonucleotides containing the binding sites for C/EBPα were purchased from Hokkaido System Science (Sapporo, Japan). The oligonucleotide sequences used for EMSA are listed in **Table S5**. EMSA was performed using a LightShift Chemiluminescent EMSA kit (Pierce Biotechnology). For each gel shift reaction (20 µL), a total of 20 fmol of labeled probe was combined with 4 µg of nuclear extract prepared from BeWo cells. Binding reactions were performed by incubating the samples for 20 min at room temperature. Protein-DNA complexes were separated from the free DNA probes by electrophoresis through 6% native polyacrylamide gels containing 0.5×Tris borate/EDTA. The gel was transferred to a nylon membrane (Thermo scientific, Lafayette, CO) and fixed for 15 min by use of a UV trans-illuminator (UVP Inc., Upland, CA). The biotin-labeled DNA was detected by addition of a streptavidin-horseradish peroxidase conjugate and chemiluminescent substrate. The competition assay was performed using a 250-fold amount of unlabeled probes with the reaction mixture before addition of biotin-labeled probes. For the super-shift assay, samples were further incubated with anti-C/EBPα antibody (sc-61X; Santa Cruz).

### Statistical analysis

Data from at least three independent experiments are expressed as the mean ± standard error of the mean. Standard error bars are included for all data points except for scatter plots. The differences between groups were analyzed using Student’s *t*-test when only two groups were present. The correlation analysis was performed using the Spearman’s correlation test (*Rs*). All tests performed were one-side. Data were considered significant if *P* < 0.05 (indicated by ‘*’).

## Supporting Information

Table S1 **Primers and positions for ChIP assay.**
(DOC)

Table S2 **Primers for bisulfite sequencing in the miR-328 5'-flanking region.**
(DOC)

Table S3 **Primers for construction of 5’-flanking reporter plasmids.**
(DOC)

Table S4 **siRNA sequences for knock-down assay.**
(DOC)

Table S5 **3’-biotin-labeled oligonucleotides for EMSA.**
(DOC)
