# Supplementary material for: Association between DNA Methylation in the miR-328 5’-Flanking Region and Inter-individual Differences in miR-328 and BCRP Expression in Human Placenta
Source: PLoS One. 2013 Aug 21;8(8):e72906. doi: 10.1371/journal.pone.0072906 (PMC3749162; doi:10.1371/journal.pone.0072906)
Supplement: Table S1 — Primers and positions for ChIP assay. (DOC) [file pone.0072906.s001.doc]

**Table S1.** Primers and positions for ChIP assay.

| **ID** | **Forward** | **Reverse** | **Position** | |
| --- | --- | --- | --- | --- |
| I | GAGCCCAGGGAGCAACAAAT | ACCCCCTTCCTTTCTTCTGG | -4135 | -4053 |
| II | GGAGGCTTTTGCTATGATGGTG | CGGCCTCCCTGTAACTGTCAAT | -3710 | -3626 |
| III | GCACTGAGGGATGAAACTGA | CCTGGCCCCAATACTGTTTT | -3463 | -3379 |
| IV | GTATGGTGAGCCAAGATGGT | CGCACCTGTCTCCAATACTT | -3167 | -3071 |
| V | TGGCAGGGGGAAGTCTGAGG | ACCCCAAGCCCGTGAGGAAA | -2707 | -2637 |
| VI | CTGCCTCAGCCTCCCGAATA | TGGTGAAACCCCGTCTCTAC | -2384 | -2313 |
| VII | CAGGCTGGTCTTGAACTC | AGACCCGGTTTCTGATTA | -2286 | -2184 |
| VIII | CCTTCATGCCTCTCACCA | CCAGAAGGCTCCTGACAA | -2081 | -1999 |
| IX | CCAAGAGCCAAGCAGAC | GGCAGTGGGTAGAGGAG | -1607 | -1550 |
| X | ACACTCACCTCCCGCTTGG | GTCCCCGCTCCCTTGCTTC | -1196 | -1096 |

Positions are relative to the miR-328 start site.
