# Supplementary material for: Association between DNA Methylation in the miR-328 5’-Flanking Region and Inter-individual Differences in miR-328 and BCRP Expression in Human Placenta
Source: PLoS One. 2013 Aug 21;8(8):e72906. doi: 10.1371/journal.pone.0072906 (PMC3749162; doi:10.1371/journal.pone.0072906)
Supplement: Table S2 — Primers for bisulfite sequencing in the miR-328 5'-flanking region. (DOC) [file pone.0072906.s002.doc]

**Table S2.** Primers for bisulfite sequencing in the miR-328 5'-flanking region.

F, forward; R, reverse

| **Primer Name** |  | **Sequence** | **Length (bp)** |
| --- | --- | --- | --- |
| 1 | F | TTTTATGGTTGTTGTAGTGGTC | 445 |
|  | R | CTTACTTCCCCAAAACCTCTAT |  |
| 1s  (second PCR) | F | TTATGGTTGTTGTTGTAGTGGT | 418 |
|  | R | TTAACCTTCTCAATCAACT |  |
| 2 | F | AGGTAGAATTGGGGGTGGTAT | 368 |
|  | R | CAACCCAACCTTCTTCCTACA |  |
