# Supplementary material for: Association between DNA Methylation in the miR-328 5’-Flanking Region and Inter-individual Differences in miR-328 and BCRP Expression in Human Placenta
Source: PLoS One. 2013 Aug 21;8(8):e72906. doi: 10.1371/journal.pone.0072906 (PMC3749162; doi:10.1371/journal.pone.0072906)
Supplement: Table S3 — Primers for construction of 5’-flanking reporter plasmids. (DOC) [file pone.0072906.s003.doc]

**Table S3.** Primers for construction of 5’-flanking reporter plasmids.

| **Primer Name** | | **Sequence** | | **Length (bp)** | **Annealing Tm(**°C**)** | **Mismatch REs** |
| --- | --- | --- | --- | --- | --- | --- |
| Full-length vector  (-4230 bp) | ins1 | F | Ctttatgtggggattcagagg | 2602 | 62.1 | - |
|  |  | R | Ccttctgccttcaaacttagc |  |  | - |
|  | ins2 | F | Cccggccctgagttaggtttta | 2247 | 60.1 | - |
|  |  | R | tatgc**A**A**G**CTTtctccctgagc |  |  | *Hind*III |
| 5’-flanking reporter plasmids | -4187 | F | cctgagctcgctagcagGG**T**ACC | 4212 | 54.5 | *Kpn*I |
|  |  | R | caCTC**G**AGgctctgagacagcc |  |  | *Xho*I |
|  | -3928 | F | gcaagccacaGGT**A**CCtcaggt | 3953 | 54.5 | *Kpn*I |
|  |  | R | ccccaCTC**G**AGgctctgagac |  |  | *Xho*I |
|  | -2428 | F | agtgcagtGGT**AC**Caatctcag | 2454 | 54.5 | *Kpn*I |
|  |  | R | ccccaCTC**G**AGgctctgagac |  |  | *Xho*I |
|  | -1395 | F | ggatgGG**T**ACCttctccagc | 1413 | 54.5 | *Kpn*I |
|  |  | R | cccccaCTC**G**AGgctctgaga |  |  | *Xho*I |
|  | -482 | F | agaaaGGT**AC**Cactgagc | 500 | 55.5 | *Kpn*I |
|  |  | R | cccccaCTC**G**AGgctctgaga |  |  | *Xho*I |

Nucleotides of primer sequences for mismatch-PCR are represented in bold letters. Tm, Temperature; REs, Restriction enzymes; F, Forward; R, Reverse.
