# Supplementary material for: Association between DNA Methylation in the miR-328 5’-Flanking Region and Inter-individual Differences in miR-328 and BCRP Expression in Human Placenta
Source: PLoS One. 2013 Aug 21;8(8):e72906. doi: 10.1371/journal.pone.0072906 (PMC3749162; doi:10.1371/journal.pone.0072906)
Supplement: Table S4 — siRNA sequences for knock-down assay. (DOC) [file pone.0072906.s004.doc]

**Table S4.** siRNA sequences for knock-down assay.

| **Target gene** | **Sense** | **Antisense** | **Institution** | **siRNA ID** |
| --- | --- | --- | --- | --- |
| **C/EBPα** | GUCGGCCAGGAACUCGUCGUU | CGACGUUCCUGGCCGACUU | Sigma Genonys |  |
| **c-jun** | GGCACAGCUUAAACAGAAAtt | UUUCUGUUUAAGCUGUGUGCCac | Ambion | s7658 |
| **c-myb** | GCCGCAGCCAUUCAGAGACACUAUA | UAUAGUGUCUCUGAAUGGCUGCGGC | Invtrogen | HSS106819 |
| **GATA2** | CGACCACUCAUCAAGCCCAtt | UGGGCUUGAUGAGUGGUCGgt | Ambion | s5596 |
| **GATA1** | GAAGGAUGGUAUUCAGACUtt | AGUCUGAAUACCAUCCUUCcg | Ambion | s5593 |
| **USF1** | CUGCUGUUGUUACUACCCAtt | UGGGUAGUAACAACAGCAGct | Ambion | s14719 |
| **USF2** | CCCUUACUCUCCAAAAAUUtt | AAUUUUUGGAGAGUAAGGGtg | Ambion | s14720 |
| **TCF3** | AAAGACCUGAGGGACCGGGAGTT | CUCCCGGUCCCUCAGGUCUUUTT | Sigma Genonys |  |
