# Supplementary material for: Association between DNA Methylation in the miR-328 5’-Flanking Region and Inter-individual Differences in miR-328 and BCRP Expression in Human Placenta
Source: PLoS One. 2013 Aug 21;8(8):e72906. doi: 10.1371/journal.pone.0072906 (PMC3749162; doi:10.1371/journal.pone.0072906)
Supplement: Table S5 — 3’-biotin-labeled oligonucleotides for EMSA. (DOC) [file pone.0072906.s005.doc]

**Table S5.** 3’-biotine-labeled oligonucleotides for EMSA.

| **5' position** | **Sense** | **Antisense** | **Length (bp)** |
| --- | --- | --- | --- |
| -1182 | cttggagggggcaatgaagttgagg | cctcaacttcattgccccctccaag | 25 |
| -2445 | gagtgcagtggtgcaatctcagctc | gagctgagattgcaccactgcactc | 25 |
| -2343 | gaccacacccagctaattgttatat | atataacaattagctgggtgtggtc | 25 |
